# Evaluation of the Clinical Utility of the Bone Metastases Ensemble Trees for Survival Decision Support Platform (BMETS-DSP): A Case-Based Pilot Assessment

**DOI:** 10.1200/CCI.22.00082

**Published:** 2022-10-28

**Authors:** Sara R. Alcorn, Anna W. LaVigne, Christen R. Elledge, Jacob Fiksel, Chen Hu, Lawrence Kleinberg, Adam Levin, Thomas Smith, Zhi Cheng, Kibem Kim, Avani D. Rao, Lindsey Sloan, Brandi Page, Susan F. Stinson, K. Ranh Voong, Todd R. McNutt, Michael R. Bowers, Theodore L. DeWeese, Scott Zeger, Jean L. Wright

**Affiliations:** ^1^ Department of Radiation Oncology, University of Minnesota Medical School, Minneapolis, MN; ^2^ Department of Radiation Oncology and Molecular Radiation Sciences, Johns Hopkins School of Medicine, Baltimore, MD; ^3^ Department of Biostatistics, Johns Hopkins Bloomberg School of Public Health, Baltimore, MD; ^4^ Department of Orthopedic Surgery, Johns Hopkins School of Medicine, Baltimore, MD; ^5^ Department of Oncology, Johns Hopkins School of Medicine, Baltimore, MD

## Abstract

The Bone Metastases Ensemble Trees for Survival Decision Support Platform (BMETS-DSP) provides patient-specific survival predictions and evidence-based recommendations to guide multidisciplinary management for symptomatic bone metastases. We assessed the clinical utility of the BMETS-DSP through a pilot prepost design in a simulated clinical environment.

**METHODS:**

Ten Radiation Oncology physicians reviewed 55 patient cases at two time points: without and then with the use of BMETS-DSP. Assessment included 12-month survival estimate, confidence in and likelihood of sharing estimates with patients, and recommendations for open surgery, systemic therapy, hospice referral, and radiotherapy (RT) regimen. Paired statistics compared pre- versus post-DSP outcomes. Reported statistical significance is *P* < .05.

**RESULTS:**

Pre- versus post-DSP, overestimation of true minus estimated survival time was significantly reduced (mean difference –2.1 [standard deviation 4.1] *v* –1 month [standard deviation 3.5]). Prediction accuracy was significantly improved at cut points of < 3 (72 *v* 79%), ≤ 6 (64 *v* 71%), and ≥ 12 months (70 *v* 81%). Median ratings of confidence in and likelihood of sharing prognosis significantly increased. Significantly greater concordance was seen in matching use of 1-fraction RT with the true survival < 3 months (70 *v* 76%) and < 10-fraction RT with the true survival < 12 months (55 *v* 62%) and appropriate use of open surgery (47% *v* 53%), without significant changes in selection of hospice referral or systemic therapy.

**CONCLUSION:**

This pilot study demonstrates that BMETS-DSP significantly improved physician survival estimation accuracy, prognostic confidence, likelihood of sharing prognosis, and use of prognosis-appropriate RT regimens in the care of symptomatic bone metastases, supporting future multi-institutional validation of the platform.

## INTRODUCTION

In the management of symptomatic bone metastases, selection of appropriate palliative radiotherapy (RT) regimens should ideally be individualized on the basis of patient-, disease-, and treatment-specific characteristics including estimated survival, cancer type, and prior, current, and potential future oncologic therapies. Yet, a number of studies have demonstrated that provider estimates of patient survival are notoriously inaccurate and overoptimistic.^1-4^ Moreover, available evidence-based and consensus guidelines do not provide clear criteria for selecting between the range of palliative RT regimens.^5-7^

**CONTEXT**

**Key Objective**
To determine if the use of the Bone Metastases Ensemble Trees for Survival Decision Support Platform (DSP) improves providers' survival predictions and adherence to evidence-based recommendations for the management of bone metastases.
**Knowledge Generated**
This pilot study used a prepost design to demonstrate that use of the Bone Metastases Ensemble Trees for Survival-DSP significantly improves accuracy of survival estimates, prognostic confidence, likelihood of sharing prognosis, and choice of prognosis-appropriate radiotherapy regimens and surgical interventions among radiation oncologists. The tool did not affect selection of prognosis-appropriate systemic therapy and hospice referral practices.
**Relevance**
A DSP can be used to guide clinical decision making in the management of symptomatic bone metastases, promoting individualized treatment approaches and the selection of evidence-based therapeutic options in this context.

To address these issues, we developed the provider-facing Bone Metastases Ensemble Trees for Survival Decision Support Platform (BMETS-DSP),^8^ which (1) collects patient-specific characteristics critical to treatment selection, (2) displays a patient-specific predicted survival curve on the basis of the validated BMETS machine learning survival model,^9-12^ and (3) provides case-specific, evidence-based recommendations for RT, open surgery, systemic therapy, and hospice referral in the care of symptomatic bone metastases.^8^ The BMETS-DSP is available on a free-access website.^13^

Although a range of decision support aids have been described in the literature, few have undergone dedicated assessment of efficacy in the clinical setting.^14^ In accordance with standards delineated by the International Patient Decision Aids Standards Collaboration, a dedicated assessment of such tools is a required metric of decision aid quality.^15^ Following a similar procedure as proposed by Cheng et al,^16^ we performed a pilot assessment of the BMETS-DSP using a prepost design in a simulated clinical environment. We aimed to provide early evidence of the clinical utility of the BMETS survival model and associated BMETS-DSP to justify its future evaluation and broader clinical application.

## METHODS

### Data Source

Case patients were derived from the previously described initial BMETS database, which included 397 patients treated to 492 symptomatic bone sites from January 2007 to January 2013.^9^ After stratifying by quartiles of actual survival time, 55 case patients were randomly selected from this population.

### Study Population

Radiation oncologists with clinical privileges at the Johns Hopkins University School of Medicine were recruited, and the first five trainee and five attending physicians to respond were selected to participate. Trainees were included to ascertain if there were differences in predictive accuracy by experience level. Moreover, the topic of palliative RT is tested on radiation oncology residency boards^17^ and is considered one of the core competencies in resident education by the Accreditation Council of Graduate Medical Education in the United States.^18^ As such, trainees may specifically benefit from the use of a decision support tool in this context.

Case histories were created, which summarized relevant data including the 27 BMETS survival model covariates,^9^ as well as additional covariates used to create individualized treatment recommendations in the BMETS-DSP^8^: prior RT, specific tumor histology, radiologic evidence of neuraxis compromise or soft tissue component at the target site, and neurologic symptoms attributable to the target lesion. To estimate predicted survival for the BMETS-DSP assessment, the BMETS model was refit using the initial training set but leaving the case patient out. Each of the 55 refitted models were then used to produce a predicted survival curve for the corresponding case patient. Individualized recommendations were generated using BMETS median predicted survival time and other patient- and disease-specific characteristics, as previously detailed.^8^

Physicians evaluated the same case histories at two time points, first without and then with the use of the BMETS-DSP output, separated by a washout period of 3-4 weeks, as per Cheng et al.^16^ The time between the start and completion of each phase of the assessment was ≤ 1 week.

This project was approved by the institutional review board at the Johns Hopkins University (IRB00198589). Informed consent was waived for case patients and obtained for participating radiation oncologists.

### Outcome Assessments

At both case assessment times, physicians answered seven identical questions regarding the case patients. Physicians were advised that there may be no single correct answer and instructed to choose their response on the basis of their clinical knowledge and practice alone at time 1 and with the assistance of the BMETS-DSP at time 2. Table 1 summarizes the assessment questions.

**TABLE 1.**
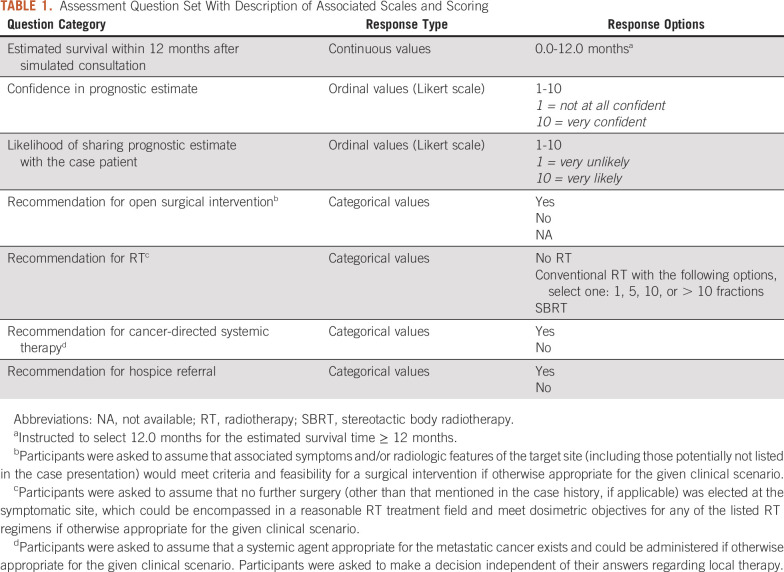
Assessment Question Set With Description of Associated Scales and Scoring

For recommended interventions, the term *appropriate* was defined as the condition in which the patient would not be excluded from the intervention on the basis of features described or implied in the case presentation. This term was meant to capture decision uncertainties including prognosis. The complete assessment form is included in the Data Supplement.

Survival estimates and intervention recommendations were also evaluated in relation to case patients' actual survival at clinically relevant binary time points of 3, 6, and 12 months. The 3-month time point (< 3 *v* ≥ 3 months) was selected to mirror the cut point used for appropriateness of spine surgery.^19^ As previously described,^8^ this cut point was also used to determine whether shorter fraction RT would be recommended. The 6-month time point (≤ 6 *v* > 6 months) corresponds to the cut point used for appropriate hospice referral.^20^ This cut point also reflects the upper range of survival for which shorter fraction RT has been tested for patients with spinal cord compression.^21^ The 12-month time (< 12 *v* ≥ 12 months) reflects a condition of relatively prolonged survival time.

### Statistical Analysis

Descriptive statistics were performed to characterize patient and disease features for case patients and to describe the participating physicians.

Physicians' estimates of survival were first assessed as a continuous variable, using the Wilcoxon signed-rank test to evaluate paired survival estimates before and after the use of the BMETS-DSP.

Physician performance in estimating survival time pre- and post-DSP was analyzed using accuracy, sensitivity, specificity, area under the receiver operative characteristics curve (AUC), and positive and negative predictive values, comparing physicians' estimates of survival versus actual survival at the clinically relevant binary time points of < 3 months, ≤ 6 months, and < 12 months. Accuracy was defined as the number of correct predictions (sum of true positives and true negatives) divided by the total number of case patients. To evaluate these performance measures, physicians' continuous survival estimates were converted to binary values of surviving versus not surviving at each time point. A true positive was defined as a correct prediction of surviving relative to actual survival at that time point. McNemar's test compared paired values of pre- and post-DSP accuracy.

Confidence in and likelihood of sharing prognostic estimates were evaluated using the Wilcoxon signed-rank test for paired ratings of these measures, pre- and post-DSP.

Appropriate selection of treatment interventions was assessed by a match between the recommendation for a given intervention and its appropriateness, as defined by evidence- or consensus-based guidelines and/or the clinically relevant binary time point. Percent of concordant matches at each assessment time was specified as the sum of [(correct recommendation for the intervention in the case where it is appropriate) plus (correct recommendation for no intervention in the case where it is not appropriate)], divided by the total number of case patients. For each intervention, appropriateness was determined using cut points and assumptions delineated in Table 2. Concordant matches for each intervention were compared pre- and post-DSP with McNemar's test, using different definitions of appropriateness when indicated.

**TABLE 2.**
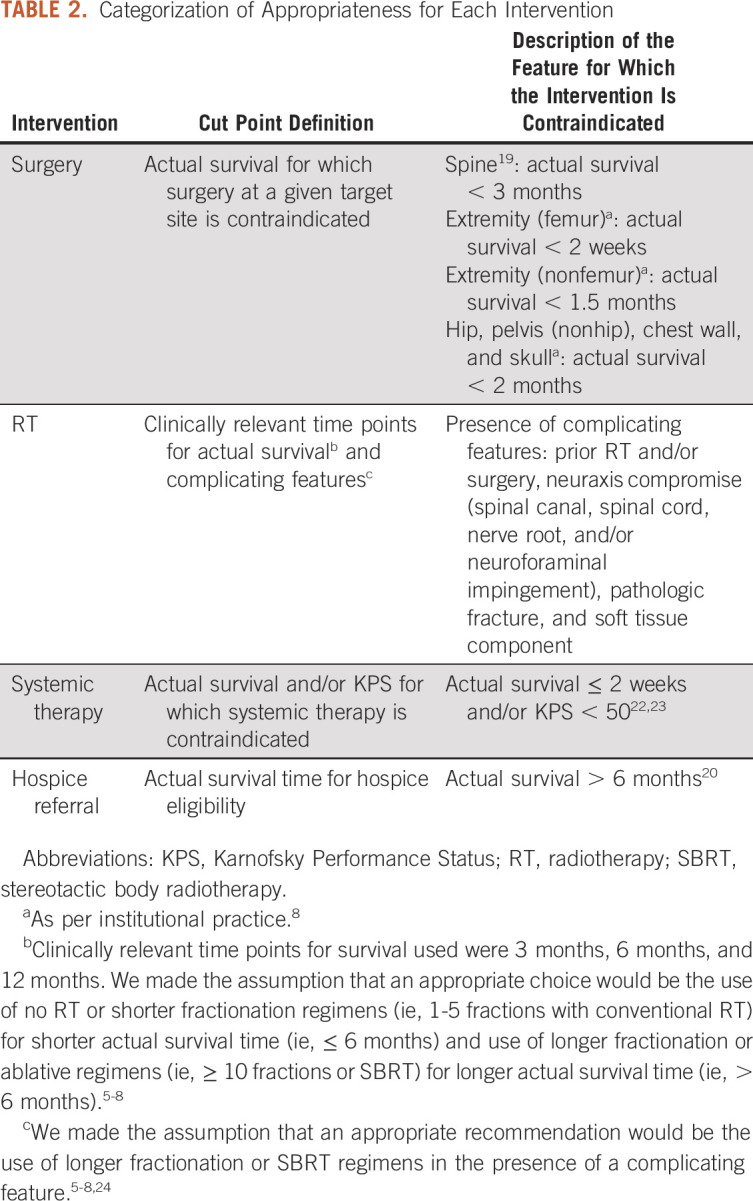
Categorization of Appropriateness for Each Intervention

All statistical tests used a two-sided α = .05 for significance testing. Confidence intervals were reported for logistic regressions as per Louis and Zeger.^25^ Statistics were performed using Stata Version 14.0 (College Station, TX).

## RESULTS

Characteristics of the included case patients are given in the Data Supplement. Distributions of key patient-, disease-, and treatment-specific factors were similar to those found in the source BMETS database.^9^

Trainee physicians (n = 5) and attending physicians (n = 5) completed medical school an average of 3.5 years (standard deviation [SD 1.3]) and 16.5 years (SD 11.6) before, respectively.

Response completion rates were 96% for pre- and post-DSP assessments.

### Estimates of Survival Time

The mean actual survival time across case patients was 5.9 (SD 4.0) months. Pre- versus post-DSP, mean physicians' survival estimates were 7.9 (SD 3.6) versus 6.9 (SD 3.7) months, respectively, *P* < .001. Use of the DSP resulted in a reduction in overestimation of actual minus estimated survival time, regardless of the participant training level, with a mean difference of –2.1 (SD 4.1) versus –1 month (SD 3.5), *P* < .001.

Table 3 displays accuracy of physicians' survival estimates at clinically relevant time points, before and after use of the BMETS-DSP. Pre-DSP accuracy was lowest when considering exact matches into survival categories and highest for the 3-month binary time point. Use of the BMETS-DSP significantly improved accuracy across all time points considered. The largest absolute increase was noted for accuracy at the 12-month binary time point, where post-DSP accuracy increased by more than 10%. Table 4 shows additional measures of physician performance for survival estimation without and with the use of the BMETS-DSP. Its use improved nearly all measures of across time points. Sensitivity, for example, was lowest at the 3-month time point but increased by more than 2-fold with the use of the DSP. Notably, specificity appeared to be least affected by the use of the DSP and was lowest at the 12-month time point. Post-DSP, AUC was improved to a satisfactory or good range for all time points, ranging from 0.66 to 0.71. Positive predictive values were increased to ≥ 0.72 post-DSP. Negative predictive values increased across all time points although it remained relatively low at 0.47 at 12 months.

**TABLE 3.**
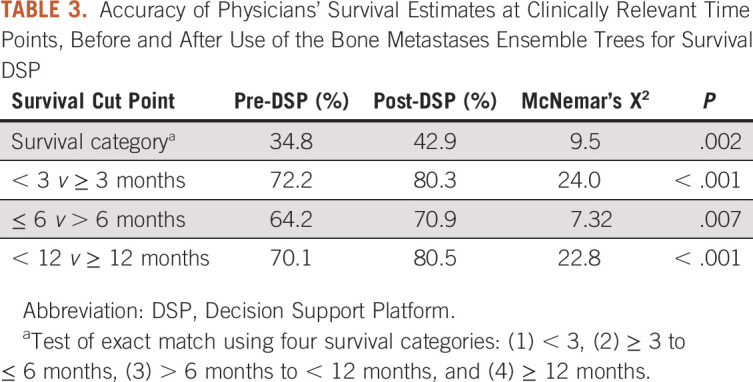
Accuracy of Physicians' Survival Estimates at Clinically Relevant Time Points, Before and After Use of the Bone Metastases Ensemble Trees for Survival DSP

**TABLE 4.**
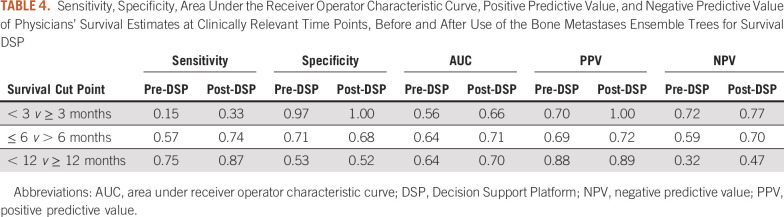
Sensitivity, Specificity, Area Under the Receiver Operator Characteristic Curve, Positive Predictive Value, and Negative Predictive Value of Physicians' Survival Estimates at Clinically Relevant Time Points, Before and After Use of the Bone Metastases Ensemble Trees for Survival DSP

### Ratings of Confidence in and Likelihood of Sharing Prognosis

Before the use of the BMETS-DSP, median ratings for both confidence with survival estimate and likelihood of sharing the survival estimate with the case patient were 6 (range, 1-10). Post-DSP, both ratings increased to a median score of 8 (range, 2-10 and 1-10, respectively). For both measures, this increase was statistically significant, *P* < .001.

### Recommendations for Appropriate Open Surgical Intervention

Seven cases were excluded for consideration because of previous open surgical intervention. After applying prognostic cutoffs specific for each treatment site, there was no clear contraindication to open surgery in 33 of 48 cases (67.4%). Before the use of the BMETS-DSP, open surgical intervention was recommended by physicians in 28.7% of cases. After the use of the BMETS-DSP, surgical intervention was recommended in 38.6% of cases. Match between prognosis-appropriate surgery status and recommendation for surgery improved pre- versus post-DSP, from 47.3% and 52.8% (McNemar's Χ^2^ = 5.24, *P* = .022).

### Recommendations for Appropriate Systemic Therapy Intervention

After applying prognostic and Karnofsky Performance Status cutoffs, there was no clear contraindication to systemic therapy in 51 of 55 cases (92.3%). Systemic therapy was recommended by physicians in 82.5% and 80.8% of cases pre- versus post-DSP, respectively. Match between appropriate use of and recommendation for systemic therapy was not significantly different pre- versus post-DSP, from 83.1% to 82.9% (McNemar's Χ^2^ = 0.01, *P* = .915).

### Recommendations for Appropriate Hospice Referral Intervention

After applying prognostic cutoffs, there was no clear contraindication to hospice referral in 29 of 55 cases (52.7%). Hospice referral was recommended by physicians in 55.8% and 53.1% of cases pre- versus post-DSP, respectively. Match between appropriate hospice referral status and recommendation for hospice referral was not significantly different pre- versus post-DSP, from 66.5% to 70.9% (McNemar's Χ^2^ = 3.18, *P* = .074).

### Recommendations for Appropriate RT Intervention

**FIG 1.**
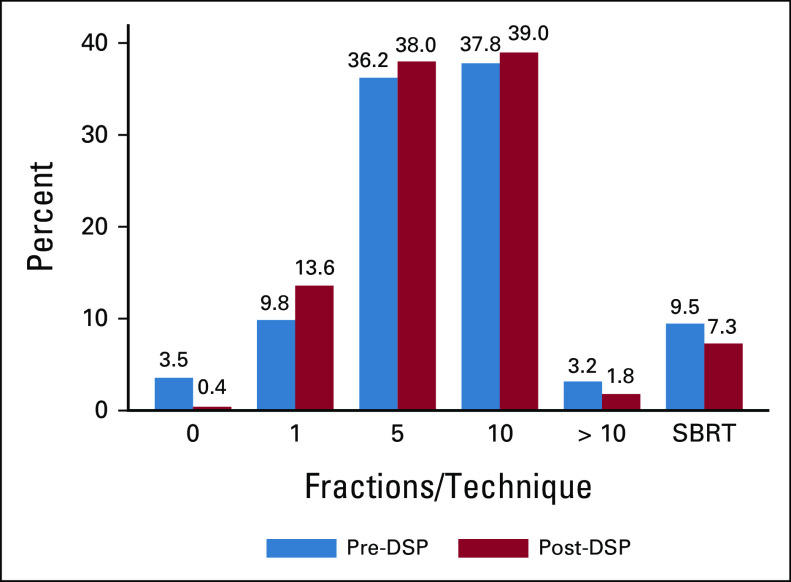
Percent of case patients for which each fractionation scheme (1 to > 10 or SBRT) was recommended, before and after the use of the Bone Metastases Ensemble Trees for Survival DSP. DSP, Decision Support Platform; SBRT, stereotactic body radiotherapy.

Figure 1 shows the percent at which each fractionation scheme was recommended, pre- and post-DSP. Treatments of > 10 fractions or with stereotactic body radiation therapy were more common pre- versus post-DSP (12.6% *v* 9.1%, respectively), whereas use of single fraction (4.9% *v* 6.8%, respectively) was more common in the post-DSP group, *P* < .001. At both assessment times, regimens using ≤ 5 fractions were selected in approximately half of cases.

Table 5 shows the percent of case patients in which there was a concordant match between the appropriate choice of a shorter fraction regimen for a patient with a lower actual survival time, evaluated at different survival times and fractionation cut points. Use of the BMETS-DSP increased concordant match of selection of ≤ 1-fraction RT for patients with the actual survival time < 3 months (McNemar’s Χ^2^ = 11.0, *P* < .001) and for patients with the actual survival time ≤ 6 months (McNemar's Χ^2^ = 4.15, *P* = .042) as well as selection of ≤ 5-fraction RT for patients with the actual survival time < 12 months (McNemar's Χ^2^ = 7.71, *P* = .006).

**TABLE 5.**
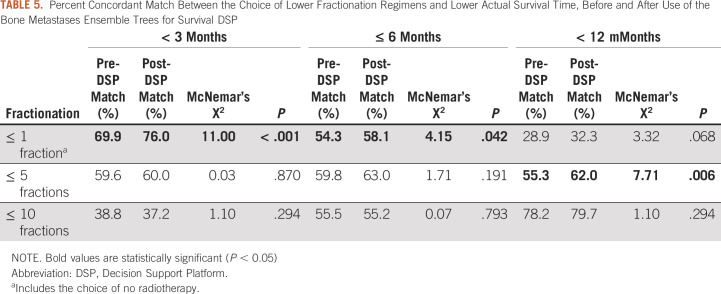
Percent Concordant Match Between the Choice of Lower Fractionation Regimens and Lower Actual Survival Time, Before and After Use of the Bone Metastases Ensemble Trees for Survival DSP

## DISCUSSION

In this pilot assessment of the BMETS-DSP, use of the decision support aid improved accuracy of physicians' survival estimates, increased confidence in and likelihood of sharing prognosis with the patient, and improved selection of prognosis- and guidelines-appropriate surgery and RT interventions. These data provide early evidence of the efficacy of the BMETS-DSP in guiding clinical decision making, with the goal of optimizing individualized care for patients with symptomatic bone metastases.

Results of the BMETS-DSP assessment confirm the trend that providers' survival estimates tend to be overoptimistic. In a systematic review regarding clinician estimates of survival for patients with cancer, nine of 12 included studies demonstrated an overestimation in survival time.^26^ Although the means by which survival estimates were measured vary between studies and limit direct comparison, our physicians' survival overestimation ratio of 1.33 (7.9 months estimated/5.9 months actual survival) falls within the range of 1.08 to 5.3 reported in other publications. Use of the BMETS-DSP reduced this overestimation ratio to 1.17, which is among the lowest ratios reported.^1^ This reduction in overestimation may be particularly important since such overoptimism is linked to low-quality, high-cost, and, low-value care in this setting.^27^ Although it could be argued that our physicians were fairly accurate in their estimations even in the pre-DSP setting, the tool also increased their stated confidence in and likelihood of sharing this prognosis. In this capacity, the tool may provide the reassurance needed to inspire action, even among good estimators.

Moreover, previous studies have shown that survival estimates may be particularly inaccurate at the extremes of survival time. Viganò et al^28^ found that physicians' sensitivity for survival prediction was lowest for patients with actual survival times ≤ 2 months. Conversely, other publications have confirmed a horizon effect—that short-term forecasts for survival and other outcomes tend to be more accurate than longer-term predictions^26^—as reflected in the results of a study of 39 patients with cancer, in which the AUC for providers' 3-month and 12-month survival predictions was 0.75 and 0.57, respectively.^29^ Given that the magnitude of improvement in survival estimates with the BMETS-DSP was greatest for discriminating between survivals at the 3- and 12-month binary time points, our tool may be an especially valuable resource for use in this setting.

Interestingly, the BMETS-DSP improved prognosis-appropriate recommendations for surgical but not for systemic therapy or hospice referral interventions. When designing the BMETS-DSP, it was not our expressed goal to allow for detailed determination of the appropriateness of surgery nor systemic therapy. Instead, the intended goal was to promote appropriate referral to medical oncology or surgical oncology colleagues should these interventions be deemed appropriate. As such, the significance (or lack thereof) of the impact of the BMETS-DSP on these interventions should be interpreted with caution. Recommendations for hospice referral were similar before and after the use of the BMETS-DSP—and notably similar to the 56% referral rate measured by retrospective review of single-institutional data.^30^ Given that physicians' accuracy for discriminating survival at the 6-month time point was improved to 70.9% at the post-DSP assessment, additional efforts should be dedicated to understanding this residual nonadherence to hospice referral guidelines.

This study is limited by its status as a pilot assessment, using a simulated clinical environment with limited sample size and nonrandomized design. Small numbers preclude the use of more advanced statistical approaches such as attempts to account for the cluster effect at the level of the provider. The primary goal of this assessment was to provide preliminary evidence of the feasibility and efficacy of the BMETS-DSP, to be used as justification for a randomized, multi-institutional study. Although its results support this goal, caution must be used in drawing extensive conclusions outside of the study's intended context. Although numerous decision support tools have been assessed in pilot studies such as this, few have been evaluated in the clinical context,^31^ where early efficacy may not translate into measurable clinical effectiveness.

Given the complex, ill-defined, and conflicting nature of guidelines available for this patient population, testing the BMETS-DSP required a number of assumptions regarding the appropriateness of the various interventions. For example, as previously reported in the development of the BMETS-DSP,^8^ shorter regimens of RT were assumed to be most appropriate for patients with more limited estimated survival on the basis of reasonable extrapolation from the literature and supported in the works of other authors.^32-34^ However, the trials of single- versus multiple-fraction palliative RT for uncomplicated symptomatic bone metastases were generally designed as noninferiority studies.^6,35^ Thus, although there is an implied benefit to the use of shorter treatment regimens in the setting of noninferiority, these data do not necessarily conclude that use of longer regimens is contraindicated. Moreover, appropriateness is a highly subjective term that is likely to vary across institutions and medical systems, potentially limiting the generalizability of these results to external users. It is also noted that the phrasing of the BMETS-DSP output indicates whether an intervention is *contraindicated* as opposed to *recommended* per se. For example, there were no contraindications to systemic therapy in 92.3% of case patients and to hospice referral in 52.7% of case patients per the criteria specified in Table 2. Given that use of systemic therapy and hospice services is generally mutually exclusive, the BMETS-DSP would not impart concrete direction to guide the provider in deciding which approach would be more appropriate in an individual case.

An additional limitation is the reliance on review of case histories as opposed to in-person patient-physician interactions. In a recent review of survival models for metastatic cancer, a subjective provider-rated variable—Karnofsky Performance Status—was typically found to be the strongest predictor of survival in this group.^9^ As such, it could be argued that the survival estimates and treatment choices garnered from our study may vary from what the physician might answer in a realistic clinical setting. However, the directionality of the impact of this potential bias is unclear. One study showed that the length of time that a physician has known a patient is linked to a reduction in prognostic accuracy—with each additional year of the patient-physician relationship resulting in a 12% increase in likelihood of prognostic error.^36^ Moreover, although some studies indicate superiority of clinician predictions over use of prognostic tools such as performance status alone,^37^ other studies show similar predictions between methods.^38^ Again, prospective evaluation of the BMETS-DSP in the clinical environment will be required to confirm its true effectiveness.

In summary, this pilot assessment of the BMETS-DSP provides preliminary evidence of its impact on improving physicians' estimates of survival and selection of prognosis-appropriate palliative RT regimens in the management of symptomatic bone metastases. These data illustrate the feasibility and efficacy of the tool, justifying more extensive assessment in a randomized, multi-institutional study with actual patients. Future iterations of the BMETS-DSP may also include additional considerations such as a patient-facing platform to enhance shared decision making and inclusion of novel palliative strategies such as the use of combined hyperthermia or ablative therapies with RT.^39,40^
